# Use of botulinum toxin in musculoskeletal pain

**DOI:** 10.12688/f1000research.2-52.v2

**Published:** 2013-07-24

**Authors:** Jasvinder A Singh

**Affiliations:** 1 Medicine Service and Center for Surgical Medical Acute Care Research and Transitions (C-SMART), Birmingham Veterans Affairs Medical Center, Birmingham AL, 35294, USA; 2 Department of Medicine at School of Medicine, and Division of Epidemiology at School of Public Health, University of Alabama, Birmingham AL, 35294, USA; 3 Department of Orthopedic Surgery, Mayo Clinic College of Medicine, Rochester MN, 55123, USA

## Abstract

Chronic musculoskeletal pain is a common cause of chronic pain, which is associated with a total cost of $635 billion per year in the U.S. Emerging evidence suggests an anti-nociceptive action of botulinum toxin, independent of its muscle paralyzing action. This review provides a summary of data from both non-randomized and randomized clinical studies of botulinum toxin in back pain and various osteoarticular conditions, including osteoarthritis, tennis elbow, low back pain and hand pain. Three randomized controlled trials (RCTs) of small sizes provide evidence of short-term efficacy of a single intra-articular injection of 100 units of botulinum toxin A (BoNT/A) for the relief of pain and the improvement of both function and quality of life in patients with chronic joint pain due to arthritis. Three RCTs studied intramuscular BoNT/A for tennis elbow with one showing a significant improvement in pain relief compared with placebo, another one showing no difference from placebo, and the third finding that pain and function improvement with BoNT/A injection were similar to those obtained with surgical release. One RCT of intramuscular BoNT/A for low back pain found improvement in pain and function compared to placebo. Single RCTs using local injections of BoNT in patients with either temporomandibular joint (TMJ) pain or plantar fasciitis found superior efficacy compared to placebo. One RCT of intramuscular BoNT/B in patients with hand pain and carpal tunnel syndrome found improvement in pain in both BoNT/B and placebo groups, but no significant difference between groups. Most evidence is based on small studies, but the use of BoNT is supported by a single, and sometimes up to three, RCTs for several chronic musculoskeletal pain conditions. This indicates that botulinum toxin may be a promising potential new treatment for chronic refractory musculoskeletal pain. Well-designed large clinical trials are needed.

## Introduction

Pain is a major public health problem. Pain was recognized as a major challenge by the Institute of Medicine (IOM) in their recent report ^1^. This report highlighted that persistent pain affects 100 million Americans with a cost of $635 billion in direct costs and lost wages. Musculoskeletal pain is the most common cause of persistent pain and is most often due to arthritis, back pain and/or local musculoskeletal conditions (such as tendinitis, bursitis, sprains), and (less commonly) injuries. In a European survey of pain prevalence in the general population, moderate to severe pain lasting 6-months or longer was reported by 19% respondents, of whom 40% (~8% of all respondents) had joint pain ^2^. Arthritis affects nearly 50 million adults in the U.S., and is projected to increase in prevalence to 67 million adults, or 25% of those aged 18 years and older, by the year 2030 ^3^. Arthritis is the leading cause of disability in adults ≥18 years in the U.S. ^4^, the third leading cause of work limitation in the U.S. ^5^, is associated with 992,100 annual hospitalizations ^6^ and 44 million annual outpatient visits ^7^. In 2003, the total cost of arthritis was $128 billion in the U.S., including $81 billion in direct medical costs and $47 billion in indirect costs (lost earnings) ^8^. Back pain is a leading cause of persistent pain and is the fifth-most-common reason for physician visits ^9, 10^. Back pain was responsible for 50% of all chronic pain problems in a European survey ^2^. In a U.S. survey, each year 15% of American adults report frequent back pain or pain lasting more than two weeks ^11^. The annual costs of lower back pain in the U.S. exceed $100 billion ^12, 13^. Treatment of chronic musculoskeletal pain secondary to joint pain and/or back pain constitutes a major challenge.

All joint structures except articular cartilage are innervated with articular nerves that contain A-delta, A-beta and C-fibers ^14– 17^. In normal healthy individuals, these nociceptors have a high threshold for excitation in response to mechanical and thermal stimuli. Normal activities such as walking, stair climbing and sports (and palpation of the joint) do not cross this threshold for nociceptor activation and therefore these activities are not associated with pain or unpleasant sensations. However, joint injury or inflammation is associated with decrease in the excitation threshold of these nociceptors. This leads to enhanced responses to both innocuous and noxious mechanical, chemical and thermal stimuli. This phenomenon is called peripheral sensitization ^18^. Chronic joint inflammation is also associated with hyperexcitability of spinal nociceptive neurons i.e., central sensitization ^19, 20^. A variety of mediators can sensitize joint nerves and nociceptors to mechanical stimuli including bradykinin, serotonin, substance P, prostaglandin E2, prostaglandin I2 and neuropeptide Y ^17^. Another contributor to joint pain and inflammation is neurogenic inflammation, which involves the release of neuropeptides from nerve terminals in response to inflammation, the release of neuropeptides from postganglionic sympathetic nerves due to sympathetic reflexes and the release of neuropeptides due to cytokine or neuropeptide-stimulation of local inflammatory cells ^21^. It is likely that these processes underlie the clinical observed signs and symptoms of pain and mechanical hyperalgesia in inflamed joints. Recent reviews describe the processes of sensitization and neurogenic inflammation and summarize our current understating of joint pain and the various mechanisms contributing to it ^17, 18^. Activation of glial cells, other immune cells and cytokines also contribute to the generation of pain ^22– 27^. The role of pain pathways, pain receptors and various contributors to both inflammatory and neuropathic pain has been summarized in recent studies and reviews ^28– 33^.

Commonly used approaches to treat musculoskeletal pain include oral therapies, intra-articular therapies, topical treatments and physical therapy. The main challenges to the use of therapies for refractory musculoskeletal pain include their limited efficacy and the risk of toxicity. Use of common oral therapies such as non-steroidal anti-inflammatory drugs (NSAIDs), opioid medications (narcotics) and analgesics (acetaminophen etc.), which are effective for some patients with chronic musculoskeletal pain, is associated with a significant risk of side effects, especially in the elderly ^34– 36^. Common side effects include: peptic ulcer disease with its complications (bleeding and perforation), renal failure and liver toxicity in patients using NSAIDs; sedation, confusion, constipation and falls in those taking narcotics; and liver toxicity in patients taking acetaminophen. Both intra-articular therapies (including the use of corticosteroids and hyaluronic acid) as well as topical preparations such as capsaicin and lidocaine have limited efficacy in patients with osteoarthritis (OA) and/or joint pain ^37– 41^. Physical therapy is effective ^42^, but feasibility remains an issue as some patients are unable to adhere to physical therapy regimens due to personal preference and/or comorbidities, the need for transportation for frequent visits and the inconvenience of frequent travel and the time commitment involved. Thus, at present, limited effective and safe therapeutic options are available for refractory musculoskeletal pain. Therefore, new therapeutic options for treatment of musculoskeletal pain are needed.

Botulinum toxin (BoNT) is one of the most potent neurotoxins and consists of a 50 kDa light chain and a 100 kDa heavy chain linked with a disulfide bond. It exists in seven serotypes, A through G. Botulinum toxin has been shown to interfere with the expression of various neuropeptides such as substance P and calcitonin gene-related protein (CGRP), which are key mediators of neurogenic inflammation ^43^. In an animal model, botulinum toxin A (BoNT/A; Onabotulinumtoxina) injections into the rat paw reduced formalin-induced paw edema, tissue glutamate release and spinal cord electrical excitations ^44^. BoNT/A inhibited stimulated substance P-release ^45, 46^ and CGRP-release ^45, 47, 48^ in models of acute and chronic inflammation. *In vitro* studies showed that BoNT/A inhibited stimulated CGRP release from rat trigeminal ganglia ^49^ and capsaicin-stimulated substance P-release from embryonic rat dorsal root ganglia neurons ^50^. In particular, BoNTs have been shown to inhibit cytokines, neuropeptides and other inflammatory mediators that play an important role in the pathophysiology of both rheumatoid arthritis and rat adjuvant arthritis (an animal model that is similar to human rheumatoid arthritis) ^51– 54^. Thus, there is pre-clinical evidence suggesting an anti-nociceptive mechanism of action of botulinum toxin. Various clinical studies support the anti-nociceptive action of botulinum toxin ^55– 57^, while some studies have found no such evidence ^58– 60^.

The objective of this systematic review was to synthesize data from both randomized and non-randomized trials to assess the safety and efficacy of botulinum toxin in osteoarticular pain.

### Search strategy for literature synthesis

In this evidence-based literature review, I performed a focused systematic review of published non-randomized (prospective and retrospective cohort studies and case series) and randomized studies of botulinum toxin for arthritis and musculoskeletal pain by performing two PubMed searches: one using the terms "botulinum toxin" and "musculoskeletal pain" and another using the terms "botulinum toxin" and "arthritis". In addition to summarizing the key studies identified from this search, I also reviewed the bibliography of key reviews to identify any studies that may have been missed using the search strategy, including our recent review of the use of botulinum toxin in osteoarticular pain ^61^. Inclusion criteria included the use of botulinum toxin, randomized or non-randomized study that included adults with musculoskeletal pain and reported clinical outcomes. Studies of myofascial pain syndromes were not included in this review, since they have been the focus of previous reviews ^62, 63^. I summarized case reports only when they provided new evidence for a particular use of botulinum toxin. In the sections below, I summarize data that provides initial evidence for the anti-nociceptive action of botulinum toxin, followed by existing evidence related to the use of botulinum toxin in various musculoskeletal pain conditions including refractory arthritis.

Recent reviews have summarized the injectable treatments for osteoarthritis ^64^, injectable treatments for knee osteoarthritis ^65^ or use of neurotoxin for the treatment of painful disorders ^66^, In this review, I provide a review of evidence regarding botulinum toxin for the treatment of musculoskeletal pain conditions.

## Results

### Refractory osteoarticular joint pain

In this section, I summarize the data from non-randomized studies followed by data from randomized controlled trials (RCTs) for the efficacy of botulinum toxin’s efficacy in refractory shoulder joint pain (1 randomized study) and refractory knee joint pain (2 randomized studies) ( Table 1).

**Table 1.** Efficacy of botulinum toxin in osteoarticular pain in randomized controlled trials.

Study	*# Patients* (male/female), *pain duration*	Study type (duration)	Groups	Mean age in years (SD)	Primary outcomes	Main result - efficacy
Singh *et al.* ^70^	N=36 (43 painful shoulders) (35 M/1 F) *8–11 years*	Double- blind RCT (1 month)	100 units of IA- BoNT/A in 1 ml of saline plus 1 ml of 2% lidocaine (treatment group; n=21), Single injection of IA-saline plus 1 ml of 2% lidocaine (placebo group; n=22)	BoNT: 72 yrs ^2^ PL: 70 yrs ^3^	Pain on VAS Drop-out due to treatment failure Short Form 36 Short form McGill Pain Shoulder pain and disability index (SPADI)	Significantly greater reduction in VAS pain scores in IA-BoNT/A (2.4) vs. placebo (0.8) group (p = 0.02) Higher proportion dropped out at 1 month from placebo (45%) than IA-BoNT/A (19%) (p = 0.13) SF-36 scores improved significantly more in IA-BoNT/A vs. placebo in 5/8 subscales (p ranging from 0.04–0.001) McGill affective dimension scores were significantly greater in IA- BoNT/A vs. placebo group (p = 0.047) Trend towards significance in SPADI disability scores (p = 0.083) No significant differences in active flexion, active abduction, SPADI pain and total and McGill sensory and total scores.
Boon *et al.* ^73^	N=60 (60 painful knees) (25 M/35 F) *6–10 years*	Double- blind RCT (8-weeks)	IA-BoNT/A at 2 doses 100 units (n=20) or 200 units (n=20) IA-corticosteroid injection with 40 mg of methylprednisolone acetate (n=20)	BoNT 100 units: 64 yrs ^13^ BoNT 200 units: 61 yrs ^9^ Corticosteroid: 61 yrs ^10^	Pain on VAS Western Ontario McMaster Osteoarthritis Index (WOMAC) 40-m walk test Short Form 36	Significant reduction in VAS pain scores with injection in each of the 3 groups, but no significant difference between groups. 2-point reduction in VAS pain scores of participants in 60% BoNT (100 units), 26% in BoNT (200 units) and 42% in corticosteroid group (p = 0.10). Significant reduction in WOMAC pain, function and stiffness scales and 40 min walk with injection in each of the 3 groups, but no significant difference between groups. SF-36 scores did not change significantly from baseline in any of the 3 groups at 8 weeks, with the exception of the SF-36 pain index, which improved in the high- dose BoNT-A group.
Mahowald *et al.* ^74^	*N=42* *Refractory* *chronic painful* *knees; pain* *duration not* *provided*	Double- blind RCT, 1-month	100 units of BoNT/A) in 1 ml of saline plus 1 ml of 2% lidocaine (treatment group, n=21) Single injection of IA-saline plus 1 ml of 2% lidocaine (placebo group, n=21)	N/A	McGill Pain VAS pain WOMAC	Changes in the McGill Pain Inventory joint pain scores were similar in the IA-BoNT/A and placebo groups at one month. (-4.7, p = 0.048 in IA-BoNT/A group and -5.6, p = 002 in the placebo group). At 3 months change in VAS pain was significant only in the IA- BoNT/A treatment group (-4.2, p = 0.002 in the IA-BoNT/A group and -4.6, p = 0.09 in the placebo group).

RCT, randomized controlled trial; BoNT, botulinum toxin; PL, placebo; SQ, subcutaneous; VAS, Visual Analog Scale; SD, standard deviation; IA, intra-articular.

### Non-randomized studies in patients with refractory joint pain

There are three non-randomized that provide evidence for the efficacy of BoNT in cases of refractory joint pain ^67– 69^. In a retrospective study, Mahowald *et al.* reported the results of intra-articular injections of botulinum toxin type A (IA-BoNT/A) in 15 shoulder and lower extremity joints in 11 patients experiencing refractory pain ^67^. All patients were receiving analgesics/NSAIDs and none had experienced relief with intra-articular corticosteroids. Patients received 25–100 units of IA-BoNT/A. There were 9 men and 2 women with an age range of 42–82 years. Joint pain was measured on a 0–10 numeric rating scale (NRS). Pain relief began within 2 weeks in most patients. A single intra-articular injection of botulinum toxin was associated with a clinically and statistically significant decrease in pain severity and improvement in function compared to baseline: lower extremity and shoulder pain decreased from 7 to 2.7 and 8.2 to 2.4, respectively; shoulder flexion improved from 68 to 113 degrees and shoulder abduction improved from 50 to 74 degrees, respectively. Timed stand tests (the time taken to stand up ten times from a sitting position) improved from 36 to 23 seconds. The duration of relief/improvement lasted 3–10 months. None of the patients reported any adverse events and no motor-sensory deficits were found on lower extremity examination after the IA-BoNT/A injection.

Another report described the long-term follow-up of these 11 patients with 15 treated joints, of which ten joints were re-injected with 30–150 units of BoNT/A intra-articularly (Botox, Allergan) at the patient’s request, when pain returned ^68^. Nine of the ten re-injections were associated with pain reduction, as seen with the first injection. Pain severity decreased from 6.6 (SD, 1.2) to 3.3 (SD, 2.7) on a 0–10 NRS, which was statistically significant (p = 0.003). Pain relief lasted 3–17 months. None of the patients reported any local or systemic adverse events related to BoNT/A. One patient experienced increased joint swelling with no increase in joint pain 3 weeks after BoNT/A injection. Another patient experienced a continued increase in joint pain after BoNT/A injection, which was relieved with a subsequent BoNT/A injection.

In another case series, 11 adults with refractory pain underwent injection of botulinum toxin type A (25–100 units; Botox, Allergan) or type B (5,000 units; Myobloc, Solstice Neurosciences) into the sacroiliac, cervical/lumbar facet or sternoclavicular joints, C-2 roots and lumbar disc ^69^. This case series comprised 9 women and 2 men with a mean age of 48 years (SD, 10 years; range, 32–68 years). Median pain scores decreased significantly after BoNT injections, with a median decrease of 3 on a 0–10 NRS pain scale (range for pain NRS reduction, 0–5; p = 0.008). Three patients experienced no change in pain severity after botulinum toxin injections, while eight experienced a decrease. No patients reported any increase in pain severity after BoNT injection. Pain reduction began within 3–5 days. Five patients received repeat injections. There was no evidence of decreasing efficacy of pain relief with repeated BoNT injections; in fact, the duration of pain relief increased with each successive treatment for 4 of the 5 patients with multiple treatments. All patients that experienced pain reduction with BoNT injections also noted improved function in activities of daily life and the range of motion in their joints. The median duration of pain relief with BoNT injections was 1.6 months longer than that seen with previous corticosteroid injections. There were no adverse events reported by the patients.

### Refractory shoulder joint pain: 1 RCT comparing IA-BoNT/A injections with IA-placebo

Singh *et al.* performed a double-blind RCT of BoNT/A (Allergan, Inc.) into glenohumeral (shoulder) joints of patients with refractory, chronic shoulder joint pain due to osteoarthritis or rheumatoid arthritis of the shoulder joint, who had all failed conservative management ^70^ ( Table 1). Patients were randomized to one of the two treatment groups - a single injection of 100 units of botulinum toxin type A (IA-BoNT/A) reconstituted in 1cc of saline plus 1 ml of 2% lidocaine (treatment group; n=21) or a single injection of IA-saline plus 1 ml of 2% lidocaine (placebo group; n=22). Patients were enrolled in the study if they had chronic knee pain of 4.5 or more on a 0–10 NRS pain scale for at least 6-months, evidence of radiographic OA, had a failed response to oral analgesics/anti-inflammatory drugs and IA-corticosteroid injections, and were not candidates for shoulder joint replacement surgery. Patients were excluded if they were currently using aminoglycoside antibiotics, curare-like agents, or other agents that might interfere with neuromuscular function; had shoulder joint malignancy, a prosthetic shoulder joint or planned shoulder joint surgery in the next 6 months; prior botulinum toxin injection into the index shoulder joint; had disorders of neuromuscular function including myasthenia gravis, Eaton-Lambert syndrome, amyotrophic lateral sclerosis; known allergy/sensitivity to study medications; a history of recent or ongoing alcohol or drug abuse, uncontrolled systemic disease; or were pregnant, breast feeding or planning pregnancy during the study period. The study had >80% power to detect 1.5 unit difference in pain NRS scale between intervention and placebo.

At a 1-month follow-up or later, if patients did not experience any reduction in pain severity (due to inefficacy of the treatment), they were given the option to drop out of the randomized phase of the study, and request the second unblinded injection with 100 units of IA-BoNT/A, which led to the beginning of an open-label phase. The published paper reported the results from the 1-month double-blind portion of the study ^70^.

36 patients with 43 painful shoulder joints were randomized. Mean age was >70 years, 95% were men, >85% had OA as the underlying diagnosis, mean shoulder pain duration was 8–11 years, comorbidity index was high, at least 50% had been treated each with narcotics or non-steroidal anti-inflammatory drugs (NSAIDs), and all had failed previous IA-corticosteroid injections.

Reductions in shoulder pain severity were significantly greater in the IA BoNT/A group compared to the IA placebo group at 1 month (-2.4 vs. -0.8 unit reductions NRS respectively, p = 0.014; primary outcome). 61% of patients in the IA BoNT/A group experienced clinically meaningful pain relief (defined as a 30% or 2-point reduction previously ^71, 72^) at 1 month compared with 36% in the placebo group (p = 0.22).

Several secondary outcomes were significantly better in the IA-BoNT/A versus the IA-placebo group. Quality-of-life improvements were significantly greater in the BoNT/A group versus the placebo group for five of the eight short form-36 (SF-36) subscales at 1-month, namely bodily pain, role physical, energy/vitality, role emotional and mental health (p-values ranging from 0.04 to 0.001). Improvements in quality of life exceeded the clinically meaningful threshold of 10-points for all 5 SF-36 subscales in IA-BoNT/A group versus as opposed to only 1 subscale in the IA-placebo group. McGill affective pain dimension improved significantly more in BoNT/A compared to placebo at 1-month (p = 0.047). Other secondary measures showed a trend in improvements, but were not statistically significantly different between treatment groups. The Shoulder Pain and Disability Index (SPADI) showed a trend towards greater improvement in BoNT/A versus placebo group (p = 0.0826). Treatment-failure after 1 month, defined as a drop-out from the blinded phase due to inefficacy and request for an active treatment injection, was 2.5-times higher in the placebo vs. the BoNT/A group; 45% (10/22) vs. 19% (4/21; p = 0.128) respectively. 61% in BoNT/A group experienced clinically meaningful pain relief at 1 month (a 2-point or 30% reduction in pain severity on the visual analogue pain scale (VAS)) compared to 36% in the placebo group (p = 0.22).

The change in pain severity was influenced by baseline SPADI disability. In those patients with a higher SPADI disability score (>61.3), the decrease in VAS pain ratings at 1 month was significantly greater in the BoNT/A group (a 3.6 reduction) compared with the placebo (0.5; p = 0.015). On the other hand, in patients with lower SPADI disability score (≤61.3), no significant difference was observed in VAS pain severity reduction between BoNT/A (a 1.9 reduction) and placebo groups (a 1.6 reduction; p = 0.73).

Overall adverse events were similar in IA-BoNT/A and IA-placebo groups. No significant difference in serious adverse events was noted. Common adverse events such as dry mouth, flu-like symptoms, dizziness etc. were not statistically significantly different between treatment groups in this small study, powered for efficacy, but not safety outcomes.

### Refractory knee joint pain: 2 RCTs of intra-articular BoNT/A (IA-BoNT/A)

***Comparison of IA-BoNT/A to IA-corticosteroid.*** Boon *et al.* compared IA-BoNT/A to IA-corticosteroids in patients with radiographic and clinical knee OA in an RCT ^73^ ( Table 1). In a single-center, prospective double-blind RCT, patients were randomized to one of three treatments - a single injection of 100 units (2 standard doses) of IA-BoNT/A (20 patients), 200 units (20 patients) or an IA-corticosteroid injection with 40 mg of methylprednisolone acetate (20 patients), into the painful knee joint. Patients were eligible for the study if they were adults ≥40 years that had both symptomatic knee OA with knee pain severity of ≥6 on a 10-point NRS scale that interfered with function most days of the week and Kellgren grade II or III tibiofemoral knee OA identified by plain radiographs. Patients were excluded if they had Kellgren grade I or IV tibiofemoral knee OA, inflammatory arthritis (such as rheumatoid arthritis, gout or pseudogout), reconstructive surgery on the affected knee, body mass index >35 kg/m ^2^, recent IA-corticosteroid or hyaluronic acid injection (last 3-months), a clinically unstable medical or psychiatric condition or history of neuromuscular disease, aminoglycoside or curare-like agents use or severe peripheral neuropathy.

The primary outcome was VAS (0–10) pain scores at 8-weeks post-injection (rated using a 10cm line). Secondary outcomes included lower extremity pain and function assessment using the Western Ontario McMaster Universities Arthritis Index (WOMAC), quality of life assessment using the SF-36, patient global assessment and time taken for a 40-meter self-paced walk (in seconds). The study was powered to detect a 2cm reduction in a VAS pain rating within each group compared to baseline scores. Non-responders were allowed to request a second injection at 8-weeks during face-to-face clinic visit after the blinded injection. Additional follow-up assessments were done at 12 and 26 weeks using mailed questionnaires.

Baseline characteristics were similar in the three groups. Patients had a mean age of 61–64 years, body mass index 28–31 kg/m ^2^, symptom duration ranging from 6–10 years and 50–65% were women. Patients had tried multiple modalities for knee pain relief in the past, including: 70–80% had tried IA-corticosteroid injections, 35–50% had tried IA-hyaluronic acid injections, 55–65% had tried acetaminophen, 75–90% had tried corticosteroids and 45–80% had tried physical therapy. All 60 patients (35 women and 25 men) completed the 8-week follow-up.

VAS pain scores decreased from 6.4±1.8cm to 5.4±2.3cm (p = 0.15; 22% reduction) in the IA-corticosteroid group. In the 100-unit IA-BoNT/A (low dose) group, pain severity decreased significantly from 6.6±1.9cm to 4.5±2.2cm (p = 0.01; 28% reduction); and in the 200 units (high-dose) IA-BoNT/A group decreased from 6.6±1.4cm to 5.9±2.4cm (p = 0.15; 26% reduction). A 2cm reduction in VAS pain scores, which is considered a clinically meaningful reduction in pain ^71, 72^, was reported by 42% in the IA-corticosteroid group, 60% in 100-unit IA-BoNT/A group and 26% in the 200-unit IA-BoNT/A group (p = 0.10 for comparison between three groups; differences between 100 and 200 units not significant). Secondary outcomes including WOMAC pain scores, function and stiffness and self-paced 40 meter walk time improved significantly within each of the three groups at 8-weeks, compared to baseline (p<0.05 for each; Table 1). However, there were no significant differences for WOMAC scores between groups at 8-weeks. SF-36 scales did not improve significantly in any group, except in the mental health subscale score at 8-weeks in the IA-BoNT/A 200 unit group ( Table 1). At the 8-week follow-up 65% in the IA-corticosteroid group, 75% in 100-unit IA-BoNT/A group and 70% in 200-unit IA-BoNT/A group said that they would have the treatment again. Seventeen patients withdrew from the study at the 8-week follow-up requesting re-injection and an additional 11 patients did not complete the 6-month follow-up questionnaire. For the patients remaining in the trial, the reduction in VAS pain was sustained at 12-weeks for both 100- and 200-unit IA-BoNT/A and at 12 and 26 weeks in the 100-unit IA-BoNT/A group, but not in the IA-corticosteroid group. No deaths, anaphylactic reaction or septic arthritis were reported in any group, strength testing did not reveal any significant changes at any time-point in treatment groups and none of the common adverse events were significantly different between groups in this small study, powered for efficacy, but not safety assessments.

***Comparison of IA-BoNT/A to IA-placebo.*** In a recent review article, Mahowald *et al.* summarized the results of an RCT comparing a single injection of 100 units of BoNT/A reconstituted in 1 ml of saline plus 1 ml of 2% lidocaine (treatment group) to a single injection of IA-saline plus 1 ml of 2% lidocaine (placebo group) in patients with refractory knee pain due to knee OA ^74^ ( Table 1). Patients were enrolled in the study if they had chronic knee pain of 4.5 or more on the NRS pain scale for at least 3 months, evidence of radiographic OA, had received no benefit from oral analgesics/anti-inflammatory drugs, IA-corticosteroid or IA-hyaluronic acid injections, and were not candidates for joint replacement surgery. Exclusion criteria were the same as those stated in the shoulder RCT described in the section earlier ^75^. We considered the patient’s report of knee pain relief 5–10 minutes after the injection and/or aspiration of joint fluid as a surrogate for an accurate intra-articular placement of the needle.

Forty-two patients were randomized to either a IA-BoNT/A or IA-placebo group. At 1-month, McGill pain scores decreased significantly in both the IA-BoNT/A (4.7, p = 0.048) and IA-placebo groups (5.6, p = 0.02). However, at 3 months the decrease in pain was significant only in the IA-BoNT/A (4.2, p = 0.002), but not in the placebo group (4.6, p = 0.09).

Due to a differential treatment effect evident on scatter plots, patients were stratified by baseline NRS pain scale scores into moderate (4.5–6.9) or severe (7 or higher) pain for exploratory analyses. In the severe knee pain group, significant changes in McGill sensory, affective and total pain scores were noted in the IA-BoNT/A, but not in the placebo group ( Table 1).

### Summary and study limitations: OA knee and shoulder pain (osteoarticular pain)

In summary, three RCTs and three non-randomized studies indicate that a single intra-articular injection of botulinum toxin is associated with a clinically meaningful reduction of pain and an improvement of function in patients with refractory joint pain due to osteoarthritis or other underlying arthritic-conditions. The non-randomized studies have the limitations of bias, lack of a placebo arm and over-amplification of the treatment effect. All three randomized studies had small samples, short follow-up, and tested a single dose of botulinum toxin. Hence, it is not known if the toxin is effective for long-term pain relief and what the most optimal dose is. Also, due to small sample size, these studies could not provide data with regards to who are the best candidates for this injection. Therefore, larger studies are needed to assess the most effective dose, the long-term safety of intra-articular injections and to define as to which patients might be the best candidates for this treatment option.

## Tennis elbow

### Non-randomized studies

In an open-label case series of 14 patients with "treatment-resistant" tennis elbow, Moore and colleagues injected 20–40 units of BoNT/A into the extensor digitorum communis and found >50% pain relief in 9/14 and complete pain relief in 4/14 patients during the 6–8 month follow-up ^76^. Pain relief began by 2 weeks in 10 patients, 3 weeks in one and after 1 month in two patients.

### 3 RCTs Comparing BoNT to placebo or surgery

Hayton *et al.* studied 40 patients with refractory tennis elbow pain with a duration of over 6 months, who had experienced no pain relief from ≥1 corticosteroid injections and a full course of physiotherapy ^77^ ( Table 2). Patients were randomized to either 50 units of botulinum toxin type A (Allergan; n=19) or normal saline placebo (2 ml; n=21) injected 5cm distal to the area of maximal tenderness at the lateral epicondyle ^77^ ( Table 2). There were no statistically significant differences in pain between the botulinum toxin and placebo groups at 3 months after the injection (difference of 1.1 between groups, p = 0.54), grip strength (difference of 0.57 kg between groups, p = 0.90), or quality of life measured by the Short Form-12 physical (difference of 6.24 points between groups, p = 0.16) and mental (difference of 4.26 points between groups, p = 0.42) component summary scores. Twelve of the 18 patients in the BoNT group had a transient extensors lag of the long finger at 1-weeks that disappeared 3-months after the injection; none reported this in the placebo group. The 1-point difference in pain scores between the BoNT and placebo group in this study is similar to that noted in studies of joint pain ^70, 73, 78^, however, the larger standard deviation likely led to this difference being non-significant in this case.

**Table 2.** Efficacy outcomes of botulinum toxin in tennis elbow in randomized controlled trials.

Study	# Patients (male/ female)	Study type (duration)	Groups	Mean age in years (SD)	Mean pain duration months (SD)	Primary outcomes	Main result - efficacy
Wong *et al.* ^79^	N=60 (49 M/11 F)	RCT, Double-blind (3-months)	Single injection of 60 units of IA-BoNT/A (Dysport, n=30) Saline placebo in SQ tissue and muscle (n=30)	BoNT: 46 yrs ^9^ PL: 44 yrs ^6^	BoNT: 12 ^9^ PL: 19 ^21^	VAS (0–100 mm) at 4- and 12-weeks	BoNT: VAS pain 25.3 at 4 weeks; 23.5 at 12 weeks PL: VAS pain 50.5 at 4 weeks; 43.5 at 12 weeks Differences significant at both 4 weeks (p<0.0001) and 12 weeks (p = 0.0006)
Keizer *et al.* ^80^	N=40 (19 M/21 F)	Randomized, not blinded, (24-months)	Surgical release (n=20) 1–2 injections of 30–40 units BoNT (n=20)	All patients: 43 yrs (range, 25–72 yrs)	All patients: 11 (range, 6–48)	Pain, range of motion, sick leave, Modified scoring system of pain, function, tenderness, and satisfaction	No difference in pain between groups Range of motion significantly better in BoNT compared to surgery group at 3 and 6 mths; no difference at 12 or 24 months. Sick leave lower in surgery group versus BoNT group at 3 months (p = 0.01), but no difference at 6, 12 and 24 months Overall score was similar in two groups at 3, 6, 12 and 24 mths
Hayton *et al.* ^77^	N=40 (21 M/19 F)	Double- blind RCT (3-months)	One 50 unit injection of BoNT/A (n=19) Saline placebo intramuscular 5cm distal to area of maximum tenderness (n=21)	All patients: 47 yrs (range, 35–71 yrs)	All patients: 11 (range, 6–48)	Pain, grip strength, Short Form-12	Differences in pain scores were not significant at 3 months No significant differences in grip strength or SF-12 scores between groups at 3 months
Lin *et al.* ^81^	N=19 elbows in 16 patients (9 M/8 F)	Double- blind RCT (3-months)	One 50 unit injection of BoNT/A (n=8) Coticosteroid injection (40 mg triamcinolone acetonide)_ into extensor carpi radialis brevis muscle near the common origin of wrist and finger extensors (n=9)	BoNT: Mean 45.9 (7.8) years Corticosteroid: 44.6 (11.0) years	Symptoms <3-months: BoNT: 8 Corticosteroid: 8	Visual Analog Scale pain, grip strength, and World Health Organization Quality of Life Brief Questionnaire	Corticosteroid injection was significantly superior to Botulinum toxin injection for improvement in pain scores at 4-weeks. Grip strength was lower in botulinum toxin injection versus corticosteroid injection No significant differences in SF- 12 scores between groups.
Placzek *et al.* ^82^	N=132 patients of whom 130 completed the study (61 M/69 F)	Double- blinded RCT (18 weeks)	One 60 unit injection of BoNT/A (n=70) Saline injection (n=62)	BoNT: Mean 47.4 (8.7) years Saline: 46.9 (9.4) years	BoNT: 8 months Saline: 10 months	Pain, strength of extension of finger and wrist, grip strength, patient global assessment or response, pain medication use	Botulinum toxin was significantly better than saline in pain relief by week 2, and improvements sustained through 18-weeks Patient global assessment of satisfaction and MD global assessments were significantly better in Botulinum toxin vs. saline group. No significant differences in grip strength were noted between groups, although it improved in both groups. Extension of third finger was decreased significantly in botulinum toxin group at 2 weeks, persisted till 14 weeks, but ceased at 18 weeks. No other significant differences in side effects were noted between groups.

RCT, randomized controlled trial; BoNT, botulinum toxin; PL, placebo; SQ, subcutaneous; VAS, Visual Analog Scale; SD, standard deviation.

In another study by Wong *et al.* 60 patients experiencing tennis elbow pain for 3 months or longer were randomized to either a single injection of 60 units of botulinum toxin A (Dysport; Ipsen) or saline placebo injections into soft tissue and muscle 1cm from the lateral epicondyle ^79^ ( Table 2). Patients were treatment-naive with no prior local injections. Patients were followed for 12 weeks in a double-blinded multicenter study. The mean age was 45 years, 49 were women and symptom duration was between 12 and 19 months in the two groups. Pain on a VAS scale (0–100mm) decreased significantly more in the active treatment group (65.5mm at baseline to 25.3mm at week 4 and 23.5mm at week 12) than placebo (66.2mm at baseline to 50.5mm at week 4 and 43.5mm at week 12). The differences between groups were statistically significant at both week 4 (p<0.001) and week 12 (p = 0.006). Mild weakness in finger extension at 4 weeks was seen in 10 patients in the BoNT/A group versus 6 patients in the saline group. Four patients in the Botulinum group had paresis of the fingers at 4 weeks (in one patient this persisted to week 12) compared to none in the placebo group, but grip strength was similar in both groups at the two time points.

Another randomized study compared 1 to 2 injections of 30–40 units of Botulinum toxin type A (Allergan Inc.) into the wrist extensor with the surgical release of the extensor origin of the extensor carpi radialis brevis tendon ^80^ ( Table 2). Forty patients with refractory chronic tennis elbow pain (average duration of symptoms ~10 months) were randomized, 20 to each treatment. The mean age of the patients was 43 years, average symptom duration was 11 months (range 6–48 months), and 21 were women. They found no differences between the groups with regards to pain and grip strength up to 2 years of follow-up, while minor differences were noted at shorter follow-up periods. At 1 year, 65% of patients in the Botulinum toxin group and 75% in the operative group had good to excellent results (based on a validated composite scale with pain and patient satisfaction items). Limitations of the study included the lack of description of outcomes and the lack of *a priori* designation of outcomes as primary versus secondary.

In a double-blinded RCT, Lin *et al.* compared botulinum type A to corticosteroid injection for the treatment of acute and subacute tennis elbow of 16 patients with 19 affected elbows ^81^. Compared to corticosteroid, botulinum type A injection was associated with less pain reduction (p = 0.02) but a greater decrease in grip strength (p = 0.01) at 4-weeks post-injection. With the progression of time since injection, the amount of pain reduction increased in the botulinum toxin group, but decreased in corticosteroid group, though this difference was not statistically significant. No significant differences between groups were noted in quality of life. Thus, corticosteroid was superior to botulinum toxin type A in relieving pain in tennis elbow at 4-weeks after injection.

A double-blinded RCT comparing a single injection of 60 units of botulinum toxin A (Dysport) compared to saline was performed in 132 patients with chronic tennis elbow in a multicenter study ^82^. The site of injection was the painful origin of the forearm extensor muscles and patients were followed up to 18 weeks. Compared to placebo, patients in the botulinum toxin A group had a significant improvement in pain as early as the second week after injection (p = 0.003). Both subjective general assessment of response to injection as well as blinded physician assessments also showed improvement in botulinum toxin A group, compared with the placebo group, both at six weeks (p = 0.001) and at 18 weeks (p = 0.001). Both groups experienced a consistent increase in fist closure strength, but there was no significant difference between groups. The extension of the third finger was observed to be significantly weakened at two weeks in the botulinum toxin A group and not saline control group, but this complication had completely resolved at 18 weeks.

### Summary and study limitations: tennis elbow

In summary, results from five RCTs of patients with tennis elbow indicate that botulinum toxin may be effective (two of the three RCTs were positive; three RCTs were negative but had a small sample size), in at least some patients with tennis elbow; however, given that 3 of the 5 trials had negative results, this needs further study. An important observation was that the studies with the largest sample sizes of 60 and 132 was positive, compared to the three negative studies that had smaller sample sizes (40, 40 and 16). A smaller sample size may have made the study underpowered and led to negative results. A short follow up period is another limitation of these RCTs. The studies differed in patient population (patients with non-refractory vs. refractory disease), duration of disease (>3 months vs. >6 months vs. 10 months), site of injection (1cm vs. 5cm from the lateral epicondyle or direct into the wrist extensor) and the dosage of preparation used (60 units of Dysport vs. 50 units of Botox vs. 30–40 unit injections of Botox) in the three RCTs respectively.

## Temporomandibular joint (TMJ) pain

### Non-randomized studies

In an open-label study of 41 patients with painful hyperactivity of the masticatory muscles, 200 units of BoNT/A (Dysport) was injected intramuscularly, with 8 conducted under electromyographic guidance) ^83^. The patients in this study had not gained pain relief from conservative treatment after 3 to 12 months of symptom duration. In 29 cases, only one treatment was administered. The majority of the injections were administered intraorally. Patients were observed for up to 12 months. 80% of patients reported an improvement in pain. The mean pain severity decreased from 6.4 to 3.5 on the 0–10cm VAS scale. Thirteen patients experienced a "major improvement" as evident by the disappearance of pain. Relief lasted 3–12 months during the observation period for most individuals; only 7 patients requested re-injection. One patient experienced temporary speech impairment and swallowing difficulty post-injection, which had completely reversed after 2–5 weeks.

In an open-label study, 46 patients who had experienced TMJ pain for a median duration of 96 months were injected with 50 units of botulinum toxin A in each masseter muscle and 25 units in each temporalis muscles under electromyographic guidance ^84^. Patients were followed up to 8 weeks. The mean age was 41 years and 39 were women. Comparing pre-injection to 8-week post-injection assessments, a significant improvement was noted in the pain VAS from a median of 8 to 5, functional disability index scores dropped from 5.3 to 3.9mm, tenderness to palpation reduced from 15.5 to 6mm and jaw opening extent improved from 29.5 to 34.5 mm (p<0.05 for assessments). No subjects reported worsening of their condition or any side effects after the injection.

In another study, 15 patients with temporomandibular disorder received 150 units of BoNT/A with 50 units in each masseter and 25 units in each temporalis muscle bilaterally ^85^. The mean age was 39 years and 13 were women with a mean symptom duration of 10 years. Compared to pre-injection, at 8 weeks post-injection, a significant improvement was noted in patients; mean pain VAS scores dropped from 7.3 to 4, functional disability index scores reduced from 5.5 to 3.1, tenderness to palpation scores improved from 17 to 7.6 and jaw opening extent increase from 27 to 34 mm (p<0.05 for assessments).

### One RCT in patients with facial pain and temporomandibular disorder

In a single-blinded randomized study, 90 patients with chronic facial pain caused by hyperactivity of the masticatory muscles, received intramuscular injections of 35 mouse units (based on the amount required for a lethal dose in mice) of botulinum toxin A (Allergan; n=60) or placebo (n=30) in both masticatory muscle ^86^ ( Table 3). The patients in the study had experienced no relief from conservative treatment after 3 to 34 months of use. A significantly greater reduction in 0–10 VAS pain scores was noted in the Botulinum toxin (a 3.2 unit decrease) compared with the placebo group (a 0.4 unit decrease) (p<0.01) was found at a follow-up conducted between 1–3 months. A greater proportion of patients in the BoNT/A group had ≥2-point improvement in VAS pain scores during the follow-up. One patient experienced temporary paralysis of facial expression muscles and swallowing difficulty post-injection, which resolved after 4 weeks. No speech impairment or systemic botulism was reported.

**Table 3.** Efficacy outcomes for botulinum toxin in temporomandibular disorder, low back pain, plantar fasciitis and carpal tunnel syndrome in randomized controlled trials.

Study	# Patients (male/female)	Study type (duration)	Groups	Mean age in years (range)	Mean pain duration in months	Primary outcome	Main result - efficacy
**Facial pain and temporomandibular disorder**							
Von Lindern *et al.* ^86^	N=90	Double- blind RCT (1–3 months)	IA-BoNT/A 35 units (n=60) Placebo injected on each side of masticatory muscle (n=30)	No information provided	Failed conservative treatment for 3–34 months	Pain VAS	Reduction in VAS pain at 1–3 months post-injection significantly greater in BoNT versus placebo (p<0.01) Greater proportion with a ≥2-point reduction in VAS pain in BoNT versus placebo: ~76% versus 10% (no statistical comparisons)
**Low back pain**							
Foster *et al.* ^89^	N=31 (15 M/16 F)	Double- blind RCT (2-months)	200 units IA- BoNT/A (n=15) Placebo injected into 5 lumbar or lumbosacral sites on more painful side (n=16)	BoNT: 47 yrs (20–73) PL: 46 yrs (21–65)	BoNT: 72 (range, 6–120) PL: 96 (range, 12–360)	Pain VAS Oswestry Back Pain Inventory	73% (11/15) patients in IA- BoNT/A group had ≥50% reduction in VAS pain compared to 25% (4/16) in placebo group at 3 wks (p = 0.012) 60% in BoNT versus 13% in Placebo had ≥50% reduction in VAS pain at 8-wks (p = 0.009) Improvement in Oswestry scores were seen in 67% of BoNT and 19% of placebo-treated patients at 8-wks (p = 0.011)
DeAndreas *et al.* ^96^	N=27 (8 M/20 F)	Double- blind RCT (2-months)	50 units IA- BoNT/A (n=27) Saline (n=13) or Bupivacaine (n=14) on the opposite side as BoNT/A	All: 51 ± 12 years	No information provided	Pain Hospital Anxiety and Depression scale [HAD-A and HAD-D], Lattinen test, a questionnaire of pain severity and its impact on daily living Oswestry questionnaire Spielberger State-Trait Anxiety Index	No difference in pain severity between groups at any follow-up time (p>0.05) No difference in Lattinen score (p = 0.011), Oswestry score or HAD-A and HAD-D cores between groups.
Fishman *et al.* ^90^	N=87; 67 completed study (5 bilateral) (21M/46 F)	Double- blind RCT (12 weeks)	200 units IA- BoNT/A (n=21) Triamcinolone + lidocaine (n=31) Placebo (n=15)	All: 57.4 ± 13.4 years	No information provided	Pain	Significant difference in proportion of patients with 50% ore more improvement in pain severity at each of the last 2 visits between BoNT/A and placebo (p = 0.001) and triamcinolone/lidocaine (p = 0.04)
**Plantar fasciitis**							
Babcock *et al.* ^92^	N=27 (43 feet) (9M/18F)	Double- blind RCT (2-months)	70 Units BoNT A (n=22) Placebo (40 units on medial aspect of the heel and 30 units in the foot arch (n=21)	All: Median age, 44 (range, 21–65)	No information provided	Pain VAS (0–10cm) Pressure algometry Maryland Foot Score (0–100) Pain relief VAS (0–10cm)	At 3 weeks, improvements were significantly higher for BoNT versus placebo: pain VAS - 2.7 (39% decrease) vs. 4.7 (p<0.004); Maryland Foot score - 72 (34%) vs. 49 (p = 0.001); pressure algometry - 2.7 (40% increase) vs. 1.8 (p = 0.003); and pain improvement scale, 4.8 vs. 0.6 (p<0.005) At 8 weeks, improvements were significantly higher for BoNT versus placebo: pain VAS - 1.6 (56% decrease) vs. 4.4 (p<0.005); Maryland Foot score - 81 (47%) vs. 54 (p = 0.001); pressure algometry - 2.8 (56% increase) vs. 1.8 (p = 0.003); and pain improvement scale, 5.0 vs. 1.2 (p<0.005)
**Hand pain and carpal tunnel syndrome**							
Breuer *et al.* ^93^	N=20	RCT, Double-blind, 3-months	2,500 units BoNT-B (n=11) Placebo injected into 3 hypothenar muscles in carpal tunnel (n=9)	No information provided	No information provided	NRS Pain WHYMPI	Pain scores, pain related sleep disturbances and WHYMPI scores improved in both groups at follow- up, but didn’t significantly differ between groups. At 6 weeks, 8/10 (80%) BoNT patients versus 6/9 (67%) placebo patients had a clinically meaningful reduction of pain VAS scores (30% or 2-point reduction). At 13 weeks 2/7 (29%) in the BoNT group versus 4/9 (44%) in the placebo group experienced a 30%/2-point reduction in VAS pain scores.

RCT, randomized controlled trial; NRS, Numeric Rating Scale; VAS, Visual analog scale; WHYMPI, West Haven-Yale Multidimensional Pain Inventory.

### Summary and study limitations: temporomandibular pain disorders

In summary, the evidence for efficacy of intramuscular injections of botulinum toxin for TMJ pain are based on non-randomized studies and one single blind study where the nature of the blinding (patient- or physician-blinded) as well as the time-point for outcome measurement were not described. Botulinum toxin may induce short-term pain relief in cases of temporomandibular disease. The results of this study are also limited by the short follow-up duration. Thus, more RCTs of botulinum toxin in TMJ disorders are needed to support its efficacy.

## Low back pain

### Non-randomized study

Ney *et al.* reported the results of injection of 200–500 units of botulinum toxin A intramuscularly in up to 4–5 trigger points per each side of the back from L2 to S1 in 60 patients with chronic low back pain in an open-label prospective study ^87^. The mean age was 47 years, mean disease duration was of 9 years, 18 were women and 62% had concurrent radicular pain. A positive response was defined by the occurrence of 2 out of the following 3 criteria: a ≥50% improvement in pain VAS scores, an improvement of 2 or more grades in the pain and functional subsets of the Oswestry Low Back Pain Questionnaire, and a ≥30% increase in the number of pain-free days from baseline. 58% of patients responded positively at 2 months and of these 17% still reported improvement at 4 months of follow-up. Most patients with improvements at 2 months reported that the beneficial effects weaned off by 4 months. 18 of the 19 patients that received re-injection reported beneficial response at a 2 month follow up. Two patients reported a mild, flu-like reaction lasting 3–5 days. None of the patients reported any muscle weakness.

### 3 RCT in patients with low back pain

A randomized study by Liu *et al.* was in Chinese and as a translation was not available, therefore, I could not include a full assessment this review ^88^. This 2-month study included 27 patients randomized to acupoint injection of botulinum toxin A into tender points at the third lumbar transverse process and an acupuncture group treated with acupuncture at the same points as in the acupoint-injection group.

Foster and colleagues compared 200 units of Botulinum toxin A (Allergan) injected intramuscular paravertebrally from L1-5/L2-S1 (n=15) to placebo (n=16) in 31 patients with chronic back pain of ≥6 month duration ^89^ ( Table 3). 40 units/site were injected at five lumbar paravertebral levels on the side of maximum discomfort. The mean age was 46 years, 16 were women and the mean pain duration was 6 years (with a range of 0.5–30 years). The number of individuals experiencing greater than 50% reduction in VAS pain scores from baseline was significantly higher in the botulinum toxin group compared with placebo: 73% vs. 25% at 3 weeks (p = 0.012), and 60% vs. 13% at 8-weeks (p = 0.009). The Oswestry Low Back Pain Questionnaire score improved significantly more patients in patients receiving Botox (67%) compared with those receiving placebo (19%, p = 0.011). No patient reported worsening of pain or function after the BoNT injection, but two patients reported worsening of pain after placebo injections.

DeAndreas *et al.* compared the efficacy of botulinum toxin A to control (saline or bupivacaine 0.25%) in relieving myofascial pain in patients experiencing mechanical low back pain due to bilateral myofascial pain syndrome involving the iliopsoas and/or the quadratus lumborum muscles ^90^. 27 patients who were enrolled in the study received a bilateral, fluoroscopically guided injection in the affected muscle(s) to randomly deliver botulinum toxin A to one side of the low back and a control drug in the opposite side. Botulinum toxin A injection did not significantly reduce pain more than saline or bupivacaine control in the contralateral side. No significant differences between groups were noted in improvement of patients’ daily life activities or psychologic status.

A double-blinded randomized study by Fishman *et al.* evaluated the efficacy of botulinum toxin A injections used in conjunction with physical therapy for the treatment of back pain due to piriformis syndrome in a 12-week study ^90^. 67 patients received 72 injections (5 bilateral) and were assigned to one of the three groups, botulinum toxin A, triamcinolone + lidocaine or saline placebo. All patients received therapy. Patients injected with botulinum toxin A experienced more pain relief than patients receiving lidocaine with corticosteroid (p = 0.04) or saline placebo (p = 0.001).

### Summary and study limitations: low back pain

In summary, intramuscular injections of botulinum toxin in patients with chronic back pain seemed to provide short-term benefits of pain reduction. This is based on one non-randomized study and two RCTs in patients with chronic low back pain. Both RCTs had a small sample size and included patients with chronic symptoms. Pain improvement was noted in both RCTs and function, measured in one study, also improved. The effects were not assessed at long-term follow-up, i.e., at 6-months or longer. In addition, in one study all groups received physical therapy, and therefore the effects in the intervention arm may be attributable to an interaction between therapy and botulinum toxin. More evidence is needed to examine the utility of botulinum toxin in the treatment of back pain.

## Plantar fasciitis

### Non-randomized study

Placzek *et al.* reported a case series of 9 patients with chronic plantar fasciitis, who were injected with 200 units of botulinum toxin A (Dysport) in the plantar fascia ^91^. Patients were followed up to one year after the injection. Statistically significant pain relief began two weeks after the injection (from 4.2 to 1.9 on a 0–10 VAS pain scale, p = 0.012) and persisted for the 52 weeks of follow up (from 4.2 to 0.4, p = 0.043). Muscle weakness or systemic effects were not seen.

### One RCT in patients with heel pain and plantar fasciitis

Babcock and colleagues compared 70 units of Botulinum toxin A injected into the plantar fascia at two sites per foot to a placebo in 27 patients (43 feet in total) who had experienced chronic refractory plantar fasciitis for 6 months or more, and who had failed to respond to conventional therapies except surgery or extracorporeal shock therapy ^92^ ( Table 3). In this study, 22 feet were randomized to BoNT/A and 21 to placebo. The median age was 44 years and 18 patients (12 with bilateral and six with unilateral plantar fascia) were women. Of the sixteen bilateral patients (male and female), 12 improved on all measures in the BoNT/A-treated foot and only one improved in the placebo-treated foot. Both pain and pain relief were measured on separate 0–10cm VAS scales. Compared to the placebo group, the Botulinum toxin group had significantly improved VAS pain relief scores (4.75cm vs. 0.6cm at 3 weeks and 4.95cm vs. 1.2cm at 8 weeks, p<0.005 for both), significantly lower VAS pain scores (2.7cm vs. 4.7cm at 3 weeks and 1.6cm vs. 4.4cm at 8 weeks, p<0.005 for both), significantly better foot function as assessed by the Maryland Foot Score (100-point scale) (72 vs. 49 at 3 weeks and 81 vs. 54 at 8 weeks, p<0.001 for both) and less muscle tenderness at plantar fascia insertion as assessed by a pressure algometry response (2.7 vs. 1.8 at 3 weeks and 2.8 vs. 1.8 at 8 weeks, p<0.003 for both). No complications were reported.

### Summary and study limitations: plantar fasciitis

In summary, the evidence is based on one non-randomized study and one RCT. Pain and function both improved short-term in botulinum toxin compared to placebo at 3- and 8-week follow-up in RCT and pain improved up to 1-year follow-up in the non-randomized study. While the data seems promising, more RCTs are needed and evidence of efficacy at longer-term is needed to assess whether botulinum toxin can provide a longer-term relief in patients with plantar fasciitis.

## Hand pain and carpal tunnel syndrome

Breuer and colleagues randomized 20 patients with carpal tunnel syndrome with associated hand pain to receive electromyographically guided placebo or botulinum toxin B injections in three hypothenar muscles anatomically attached to the carpal tunnel ^93^ ( Table 3). The dose used in 18 of the 20 patients due to modified protocol was 2,500 units, since the first two patients reported weakness and stiffness of the fourth and fifth fingers with higher doses of 5000 and 7500 units. During the 13-week trial, significant decreases in pain outcomes and improvements in function were noted in both placebo and the BoNT/A groups compared to the baseline, but there were no significant differences between the two groups. The unblinding of the first two patients and variation in the BoNT/A dose during the trial make interpretation of findings from this study difficult.

## Anterior knee pain

One non-randomized study provides evidence for the use of botulinum toxin in cases of anterior knee pain. Singer *et al.* injected 300 to 500 units of botulinum toxin A (Dysport) into the vastus lateralis muscle in 8 women with chronic anterior knee pain of more than 6 months duration who had failed to respond to conservative management, e.g. patello-femoral bracing or taping ^94^ ( Table 3). The injection was followed by a 12-week home exercise program to strengthen the vastus medialis. The mean age was 29 years and mean symptom duration was 5 years (range, 1–19 years). Patients reported a decrease in knee pain (individual changes were shown in the article, not means), an increase in function with an improvement in the mean force production in the affected limb from 22.7 kg to 24.3 kg) and an improvement in the mean time taken to ascend and descend a flight of 11 stairs from 12 to 10 seconds. These improvements were maintained at a 24-week follow up.

## Hip pain

One non-randomized study provides evidence for the use of botulinum toxin in hip pain. A total of 400 units of Botulinum toxin type A (Dysport) were injected into the adductor longus and the adductor magnus muscles in 39 patients with hip osteoarthritis ^95^. The mean age was 68 years (age range 41–82 years). The Harris Hip Score, a commonly used measure of hip function, increased significantly after 2, 4 and 12 weeks after the BoNT/A injection (p<0.0001). There was a significant decrease in pain 2, 4 and 12 weeks after the BoNT/A injection (p< 0.001).

## Limitations of this review

This was a focused review of randomized and non-randomized studies, not a systematic review or a meta-analysis. Therefore findings must be interpreted with caution. In particular, the summary section for various musculoskeletal pain disorders highlights the limitation of studies included. In particular, evidence for the efficacy of botulinum toxin in anterior knee pain and hip pain was based on non-randomized study data only (one study for each), and is subject to bias. Also, the evidence presented here includes both intramuscular and intra-articular injections for a variety of musculoskeletal disorders. Even though presented together, their effects may differ. The intramuscular injections may have complex effects, including the analgesic effects, effects from blocking acetylcholine release from motor nerve endings, and the elimination of muscle myofascial trigger points ^96^. On the other hand, intra-articular injections may work primarily by their effect on the analgesic pathways ^97^.

## Summary and conclusions

In this review, I have summarized studies of the use of botulinum toxin for musculoskeletal pain. Most studies used either an intra-articular or intramuscular route of administration in patients with refractory pain, who had not responded to multiple other treatment interventions. Pre-clinical laboratory evidence supports an independent anti-nociceptive mechanism of action of botulinum toxin. While there are several osteoarticular conditions for which botulinum toxin has been studied in an RCT compared to placebo, for several conditions such as low back pain, plantar fasciitis, temporomandibular disorder and carpal tunnel syndrome, the evidence is based on non-randomized data and only a single RCT. For other conditions, such as osteoarthritis of the knee/shoulder and tennis elbow data in support of its use from several RCTs and non-randomized studies was available. Most RCTs included in this review had several limitations. Most studies were single center, had a small sample size, short follow-up and in some cases, non-standardized injection techniques. The evidence for an anti-nociceptive action of botulinum toxin in osteoarticular pain is growing. Side effects seem to be mild, and in cases of muscle weakness, reversible; however, data from larger samples needs to be generated. Evidence from larger multicenter studies of longer duration that test various doses, regimens and routes of administration of botulinum toxin are needed to better define its role in management of osteoarticular pain.
