# Supplementary material for: Use of botulinum toxin in musculoskeletal pain
Source: F1000Res. 2013 Jul 24;2:52. Originally published 2013 Feb 15. [Version 2] doi: 10.12688/f1000research.2-52.v2 (PMC3968894; doi:10.12688/f1000research.2-52.v2)
Supplement: Peer review history

# Peer review: Referee response for version 2

**Published:** 2014-01-27

**Authors:** Mats Brittberg

**Affiliations:** 1 Cartilage Research Unit, University of Gothenburg, Kungsbacka, Sweden

Musculoskeletal pain is a big problem for society. There is need of new and easy performed treatment alternatives. This very comprehensive review on the treatment with botulinum toxins for different musculoskeletal pain disorders is subsequently very welcome for all that treat patients with retractable pain in bone, muscles, and joints. I am happy to recommend a further study of this well written review.

I have read this submission. I believe that I have an appropriate level of expertise to confirm that it is of an acceptable scientific standard.

# Peer review: Referee response for version 2

**Published:** 2013-08-21

**Authors:** Robert Gerwin

**Affiliations:** 1 Pain & Rehabilitation Medicine, Bethesda, MD, USA

I am now satisfied with the revised paper.

I have read this submission. I believe that I have an appropriate level of expertise to confirm that it is of an acceptable scientific standard.

# Response

**Published:** 2013-10-06

**Authors:** Jasvinder Singh

**Affiliations:** Rheumatology, Minneapolis VA Medical Center, USA

We thank Dr. Gerwin for his critique and recommendations.

# Peer review: Referee response for version 1

**Published:** 2013-03-12

**Authors:** Julia Funk

**Affiliations:** 1 Centrum für Muskuloskeletale Chirurgie, Orthopädische Universitätsklinik der Charité, Berlin, Germany

The manuscript is a decent, very detailed, and almost complete review. The title and abstract are appropriate for the content of the article and the conclusions are sensible and justified.

However, I do have two significant reservations. Unfortunately, for a complete review of the published data concerning the topic 'botulinum toxin and pain' we found two RCTs missing: Lin YC (2010) *Am J Phys Med Rehabil*, Placzek R (2007) *JBJS Am*.

Furthermore, there have been at least 3 review articles with the same topic in 2012 ( Hameed F (2012), Cheng OT (2012), Sim WS (2011)), hence by reading this manuscript, little information may be new to the informed reader.

I have read this submission. I believe that I have an appropriate level of expertise to confirm that it is of an acceptable scientific standard, however I have significant reservations, as outlined above.

# Response

**Published:** 2013-05-21

**Authors:** Jasvinder Singh

**Affiliations:** Birmingham VA Medical Center and Department of Medicine, University of Alabama, USA

We thank the reviewers, who provided great insights. The revisions made in response to their comments have improved the quality and clarity of this paper. Here are our responses to the comments from reviewer #1.   

**Reviewer comments** 

**Response to reviewers** 

Reviewer#1: **Julia Funk**, Centrum für Muskuloskeletale Chirurgie, Orthopädische Universitätsklinik der Charité, Berlin, Germany 

**Status and Report:** 12 Mar 2013     

*The manuscript is a decent, very detailed, and almost complete review. The title and abstract are appropriate for the content of the article and the conclusions are sensible and justified.*   

Thank you. 

*However, I do have two significant reservations. Unfortunately, for a complete review of the published data concerning the topic 'botulinum toxin and pain' we found two RCTs missing: Lin YC (2010) Am J Phys Med Rehabil, Placzek R (2007) JBJS Am.* 

We thank the reviewer for pointing us to the two missing studies, which have now been included in the review. 

*Furthermore, there have been at least 3 review articles with the same topic in 2012 ( Hameed F (2012), Cheng OT (2012), Sim WS (2011)), hence by reading this manuscript, little information may be new to the informed reader.* 

We thank the reviewer for pointing out these pertinent reviews. We refer to them in our paper and discuss how our review adds to what’s already been published. Both Hameed and Cheng *et al.* were focused on osteoarthritis (OA) treatments only. Our review is more comprehensive in that it includes muscucloskeletal (MSK) pain, not just knee OA or OA. 

Sim *et al.* provides a very brief overview of a few studies of botulinum toxin for any indication, without providing much detail about key studies. Thus, we believe our review adds to the already published reviews, and provides a complete perspective on the potential role of botulinum toxin in the treatment of MSK pain. 

“Recent reviews have summarized the injectable treatments for osteoarthritis (64), injectable treatments for knee osteoarthritis (65) or use of neurotoxin for the treatment of painful disorders (66), In this review, we provide a review of evidence regarding Botulinum toxin for the treatment of musculoskeletal pain conditions.”

# Peer review: Referee response for version 1

**Published:** 2013-03-11

**Authors:** Robert Gerwin

**Affiliations:** 1 Pain & Rehabilitation Medicine, Bethesda, MD, USA

This article reviews the use of botulimum toxin in the treatment of pain, rather than in the treatment of muscle contraction. This is a new area of potentially great interest and therefore the review is both timely and presents a new use of botulinum toxin to be considered. 

The drawback in the article is that it is a systematic review, not a metaanalysis and not a critical review. As such, it could be shortened by summarizing the studies with less detail. The greater detail in which the cases are described does not add a great detail, since they are simply recapitulations of the study, and do not tell tell us if the study is credible or not. I suggest that the author either be more concise in the summaries, or be more critical in assessing the studies. 

Moreover, the author includes both intra-articular injections and intramuscular injections. The intramuscular injections may have complex effects, including; analgesic effects, effects from blocking acetylcholine release from motor nerve endings, and the elimination of muscle myofascial trigger points. 

However, the review is incomplete. For example, he  discusses low back pain with injections into the lumbar paraspinal musculature, but only gives Ney and Forster as two references. He could have included the work of Gul *et al* and of DeAndres *et al*.  

The title and abstract are appropriate.  

My comments regarding the nature of his review are given above.  The conclusion is accurate and justified. There are no experimental data to discuss, since this is a review article. 

In summary, the topic is timely, the review summarizes the data from some but not all relevant studies and in that sense is not complete, and the summary does not address the credibility of the articles cited, and that is a weakness.

I have read this submission. I believe that I have an appropriate level of expertise to confirm that it is of an acceptable scientific standard, however I have significant reservations, as outlined above.

# Response

**Published:** 2013-05-21

**Authors:** Jasvinder Singh

**Affiliations:** Birmingham VA Medical Center and Department of Medicine, University of Alabama, USA

We thank the reviewers, who provided great insights. The revisions made in response to their comments have improved the quality and clarity of this paper. Here are our responses to the comments from reviewer #2.   

**Reviewer comments** 

**Response to reviewers** 

**Robert Gerwin**, Pain & Rehabilitation Medicine, Bethesda, MD, USA 

**Status and Report:** 11 Mar 2013     

*This article reviews the use of botulimum toxin in the treatment of pain, rather than in the treatment of muscle contraction. This is a new area of potentially great interest and therefore the review is both timely and presents a new use of botulinum toxin to be considered.* 

Thank you. 

*The drawback in the article is that it is a systematic review, not a metaanalysis and not a critical review. As such, it could be shortened by summarizing the studies with less detail. The greater detail in which the cases are described does not add a great detail, since they are simply recapitulations of the study, and do not tell tell us if the study is credible or not. I suggest that the author either be more concise in the summaries, or be more critical in assessing the studies.*  

We agree and have listed this as a limitation of our review. We have added an overall limitation section for our review. We have also added a section of comments related to study strengths and weaknesses of each indication to provide a more critical assessment of each study.    

 “ **Summary and Study limitations:** ***plantar fasciitis*** In summary, the evidence is based on one non-randomized study and one RCT.  Pain and function both improved short-term in botulinum toxin compared to placebo at 3- and 8-week follow-up in RCT and pain improved up to 1-year follow-up in non-randomized study.  While the data seems promising, more RCTs are needed and evidence of efficacy at longer-term is needed to assess whether botulinum toxin can provide a longer-term relief in patients with plantar fasciitis.”   

“ **Limitations of this review** This was a focused review of randomized and non-randomized studies, not a systematic review or a meta-analysis.  Therefore findings must be interpreted with caution.  In particular, the summary section for various musculoskeletal pain disorders highlights the limitation of studies included.  In particular, evidence for the efficacy of botulinum toxin in anterior knee pain and hip pain was based on non-randomized study data only (one study for each), and is subject to bias. The intramuscular injections may have complex effects, including the analgesic effects, effects from blocking acetylcholine release from motor nerve endings, and the elimination of muscle myofascial trigger points.  On the other hand, intraarticular injections may work primarily by their effect on the analgesic pathways. “   

*Moreover, the author includes both intra-articular injections and intramuscular injections. The intramuscular injections may have complex effects, including; analgesic effects, effects from blocking acetylcholine release from motor nerve endings, and the elimination of muscle myofascial trigger points. * 

We agree that intramuscular and intra-articular injections likely have different mechanisms of action.  Both types of injections are commonly used in the treatment of MSK pain, the focus of our paper. We mention the important point brought up by the reviewer in the discussion of results. 

*However, the review is incomplete. For example, he discusses low back pain with injections into the lumbar paraspinal musculature, but only gives Ney and Forster as two references. He could have included the work of Gul et al and of DeAndres et al.*   

We have now added DeAndres *et al. *and Lew *et al*. to our review. We are sorry but could not find Gul *et al*. 

*The title and abstract are appropriate. * 

Thank you. 

*My comments regarding the nature of his review are given above.* *The conclusion is accurate and justified. There are no experimental data to discuss, since this is a review article.*  

Thank you. 

*In summary, the topic is timely, the review summarizes the data from some but not all relevant studies and in that sense is not complete, and the summary does not address the credibility of the articles cited, and that is a weakness.*

We have now included the additional studies pointed out by the reviewer. We have also added statements related to the credibility of each studies for each condition (please see responses to reviewer comments in the sections above and the changes in the revised article).
